# Dynamic Responses of Photosynthetic Parameters in *Lonicera japonica* Leaves During Photosynthetic Induction Under Elevated CO_2_ Concentrations

**DOI:** 10.3390/plants15192929

**Published:** 2026-09-24

**Authors:** Erli Tian, Yongchuang Liu, Mengdi Mu, Yunmin Wei, Guoyin Li

**Affiliations:** 1 College of Life Science and Agronomy, Zhoukou Normal University, Zhoukou 466001, China; erlitian@whu.edu.cn (E.T.); 20201064@zknu.edu.cn (Y.L.); 17203791658@163.com (M.M.); 2 Key Laboratory of Plant Genetics and Molecular Breeding, Zhoukou Normal University, Zhoukou 466001, China

**Keywords:** photosynthetic induction, elevated CO_2_, *Lonicera japonica*, stomatal conductance, biochemical limitation

## Abstract

Photosynthetic induction is a critical physiological process through which plants rapidly adjust their photosynthetic machinery in response to a step increase in light intensity. While steady-state photosynthetic responses to elevated CO_2_ (eCO_2_) are well characterized, the characteristics of photosynthetic induction in honeysuckle under varying CO_2_ concentrations remain underexplored, particularly for ecologically important species such as honeysuckle (*Lonicera japonica*). In this study, we investigated the photosynthetic induction responses of *L. japonica* leaves subjected to an abrupt light transition from 100 to 1200 µmol m^−2^ s^−1^ under three CO_2_ concentrations: 420 μmol mol^−1^ (ambient, aCO_2_), 620 μmol mol^−1^ (medium, mCO_2_), and 820 μmol mol^−1^ (elevated, eCO_2_). Key photosynthetic parameters, including net photosynthetic rate (*A*), stomatal conductance (g_s_), intercellular CO_2_ concentration (C_i_), and apparent maximum Rubisco carboxylation rate (*V*′*_cmax_*), were continuously estimated throughout the induction process. Results showed that eCO_2_ significantly enhanced steady-state *A* while simultaneously reducing g_s_ and *V*′*_cmax_*, indicating that higher CO_2_ availability drives carbon gain primarily through increased substrate supply rather than enhanced enzymatic performance. Importantly, the time required to reach 90% induction state of steady-state *A* (t_A90_) and *V*′*_cmax_* (t_V′cmax90_) decreased markedly with increasing CO_2_. Time-integrated limitation analysis revealed that biochemical limitation consistently dominated the induction process regardless of CO_2_ treatment, while stomatal limitation remained uniformly low. These findings demonstrate that eCO_2_ substantially accelerated photosynthetic induction in *L. japonica*, as reflected by faster gas-exchange-derived induction parameters (e.g., t_A90_ and t_V′cmax90_), without altering the fundamental limitation hierarchy governing the induction process.

## 1. Introduction

Atmospheric CO_2_ concentrations have risen from pre-industrial levels of approximately 280 ppm to over 420 ppm at present and are projected to reach 550–900 ppm by the end of this century, depending on emission scenarios [1]. As one of the most significant global environmental changes affecting terrestrial ecosystems, this sustained CO_2_ enrichment profoundly influences plant physiology, particularly photosynthetic carbon assimilation and stomatal regulation [2,3]. Numerous Free-Air CO_2_ Enrichment (FACE) and indoor cultivation studies have consistently demonstrated that rising atmospheric CO_2_ concentrations enhance the steady-state net photosynthetic rate of plants under constant light conditions [4]. This enhancement is primarily due to the increased CO_2_ substrate availability for ribulose-1,5-bisphosphate carboxylase/oxygenase (Rubisco), alongside reduced stomatal conductance and suppressed photorespiration [5,6]. However, most studies characterizing these responses have focused strictly on steady-state conditions, leaving the dynamic photosynthetic behavior under naturally fluctuating light comparatively understudied.

In natural environments, plants rarely experience constant irradiance. Instead, they are frequently exposed to highly fluctuating light conditions, including abrupt appearances and disappearances of sunflecks, rapid changes in light intensity within canopies caused by leaf shading, and variation in light environments associated with crop canopy structural dynamics in agricultural systems [7]. When leaves transition from low to high light, photosynthetic rate does not immediately reach a new steady state but undergoes a gradual induction process. This process is primarily limited by the rate of increase in stomatal conductance and the activation rate of photosynthetic enzymes, particularly Rubisco [8,9]. The speed and efficiency of photosynthetic induction directly influence the overall photosynthetic productivity of plants, particularly in dynamic natural environments where light availability is highly variable [10]. Previous studies have shown that slow photosynthetic induction can result in substantial losses in potential daily carbon gain [11,12], highlighting the importance of understanding the mechanisms regulating photosynthetic responses under fluctuating light.

The kinetics and magnitude of photosynthetic induction are heavily modulated by environmental conditions, particularly CO_2_ availability [13]. Elevated CO_2_ (eCO_2_) has been identified as a key factor accelerating induction, primarily through enhanced Rubisco activation rates and reduced diffusional limitation [14]. A higher atmospheric CO_2_ concentration increases the concentration gradient between the air and the chloroplast, thereby alleviating substrate limitation for Rubisco even before stomata fully open [15]. Simultaneously, high CO_2_ often induces partial stomatal closure, reducing transportational water loss without proportionally limiting carbon assimilation, thereby improving plant water-use efficiency [16,17]. Previous studies have suggested that eCO_2_ may accelerate photosynthetic induction mainly by enhancing apparent Rubisco activation and reducing diffusional limitations [18]. Nevertheless, how eCO_2_ affects the temporal dynamics of stomatal and biochemical limitations during photosynthetic induction, and whether it alters their relative contributions, remains insufficiently understood.

Honeysuckle (*Lonicera japonica*)*,* a perennial climbing vine of the Caprifoliaceae family, is extensively cultivated in China for its diverse pharmacological and ecological benefits [19]. Driven by its vigorous vining habit, the plant typically forms complex canopies where leaves must constantly navigate highly erratic spatial and temporal light fluctuations in both wild and agricultural settings. The ability of honeysuckle leaves to efficiently adjust to these rapid light transitions is critical, as their dynamic photosynthetic capacity fundamentally dictates vegetative growth, flower-bud yield, and the accumulation of valuable bioactive compounds [20]. Although the continuous rise in atmospheric CO_2_ will inevitably interact with these transient light responses, physiological research on this species has been confined to static environmental conditions. Consequently, the transient kinetics of its photosynthetic induction across varying CO_2_ environments, as well as the underlying regulatory roles of stomatal versus biochemical limitations, remain entirely unexplored.

To bridge these knowledge gaps, we monitored the transient kinetics of net photosynthetic rate (*A*), stomatal conductance (g_s_), intercellular CO_2_ concentration (C_i_), and apparent maximum carboxylation rate (*V_cmax_*) during abrupt transitions from low to high light under three CO_2_ concentrations. Our analysis systematically quantified the steady-state photosynthetic traits at both light extremes, the time required to reach 90% induction state of steady-state assimilation, and the time-integrated dynamics of stomatal versus biochemical limitations. Ultimately, this work seeks to mechanistically elucidate how varying CO_2_ levels modulate photosynthetic induction, revealing the plant’s adaptive strategies to coupled rising CO_2_ and fluctuating light. These insights will provide a crucial physiological baseline for optimizing cultivation practices and assessing the ecological resilience of valuable species under future climate conditions.

## 2. Results

### 2.1. Variations in Photosynthetic Parameters of Honeysuckle Leaves During Photosynthetic Induction Under Different CO_2_ Concentrations

Following an abrupt light transition from 100 to 1200 µmol m^−2^ s^−1^, honeysuckle leaves showed rapid and consistent photosynthetic adjustments in response to the different chamber CO_2_ concentrations applied during measurement (ambient, aCO_2_; medium, mCO_2_; and elevated, eCO_2_) (Figure 1). Specifically, *A*, g_s_, and *V*′*_cmax_* all increased rapidly, reaching a steady state within approximately 300 s. Meanwhile, intercellular CO_2_ concentration (*C*_i_) experienced a transient drop before stabilizing, indicating that the demand for CO_2_ at this stage exceeded the supply provided by stomatal diffusion. As expected, eCO_2_ substantially boosted leaf photosynthetic capacity. The steady-state *A* peaked under eCO_2_, followed by mCO_2_ and aCO_2_, demonstrating a greater magnitude of induction at higher CO_2_ levels. Interestingly, steady-state g_s_ and *V*′*_cmax_* followed an inverse pattern (aCO_2_ > mCO_2_ > eCO_2_). Finally, steady-state *C*_i_ scaled directly with external treatments, stabilizing at roughly 600, 450, and 300 µmol mol^−1^ for eCO_2_, mCO_2_, and aCO_2_, respectively. This strong proportional relationship indicates that ambient CO_2_ remained the primary driver of *C*_i_.

### 2.2. Initial Photosynthetic Parameters of Honeysuckle Leaves During Low-Light Condition Under Different CO_2_ Concentrations

Photosynthetic parameters measured at the end of the low-light condition phase (100 µmol m^−2^ s^−1^), immediately before induction, established baseline photosynthetic parameters that varied in their sensitivity to CO_2_ concentrations (Figure 2). Under these light-limited conditions, neither the initial net photosynthetic rate (*A*_i_) nor initial stomatal conductance (g_si_) differed significantly across treatments, suggesting that elevated CO_2_ did not meaningfully alter baseline photosynthetic capacity or stomatal aperture. In contrast, the treatments significantly affected both initial intercellular CO_2_ concentration (*C*_ii_) and apparent maximum Rubisco carboxylation rate (*V*′*_cmax_*_i_). *C*_ii_ maintained a pronounced gradient that tracked external CO_2_ levels despite the low light. Strikingly, *V*′*_cmax_*_i_ declined as CO_2_ concentration increased, dropping from ~23 µmol m^−2^ s^−1^ under aCO_2_ to ~16 µmol m^−2^ s^−1^ under eCO_2_.

### 2.3. Steady-State Photosynthetic Parameters of Honeysuckle Leaves During High-Light Induction

At the end of the high-light phase (1200 µmol m^−2^ s^−1^), photosynthetic parameters of honeysuckle leaves diverged markedly among CO_2_ treatments (Figure 3). Steady-state net photosynthetic rate (*A*_f_) rose significantly with increasing CO_2_, climbing from ~13.5 µmol m^−2^ s^−1^ under aCO_2_ to ~18 µmol m^−2^ s^−1^ under mCO_2_ and reaching its peak under eCO_2_. This suggests that elevated CO_2_ strongly enhances photosynthetic capacity under saturating light. Conversely, steady-state stomatal conductance (g_sf_) exhibited no significant difference between CO_2_ treatments. Steady-state intercellular CO_2_ concentration (C_if_) remained strictly driven by the external environment. C_if_ rose sequentially from ~280 µmol mol^−1^ under aCO_2_ to ~580 µmol mol^−1^ under eCO_2_, indicating that external CO_2_ availability outweighed the effects of reduced stomatal conductance. Finally, the steady-state maximum Rubisco carboxylation rate (*V*′*_cmax_*_f_) was progressively inhibited by rising CO_2_ levels. Values dropped significantly from ~80 µmol m^−2^ s^−1^ under aCO_2_ to ~73 µmol m^−2^ s^−1^ under mCO_2_, and reached a low of ~63 µmol m^−2^ s^−1^ under eCO_2_.

### 2.4. Dynamics of the Photosynthetic Induction Process in Honeysuckle Leaves Under Different CO_2_ Treatments

During photosynthetic induction, the time required for net photosynthetic rate and Rubisco carboxylation activity to reach 90% induction state of their steady-state values was sensitive to CO_2_ availability (Figure 4). The exponential decay functions provided good fits for the aCO_2_ and mCO_2_ treatments, with R^2^ values ranging from 0.922 to 0.950 for A and from 0.928 to 0.949 for *V*′*_cmax_*, indicating excellent fits. However, under eCO_2_, the fits were considerably poorer, with R^2^ = 0.785 for A and 0.724 for *V*′*_cmax_* (Figure S2). Overall, elevated CO_2_ substantially accelerated photosynthetic induction. Specifically, the induction time for net photosynthetic rate (t_A90_) dropped progressively from ~194 s under aCO_2_ to ~117 s under eCO_2_. A closely parallel pattern emerged for the induction time of apparent maximum Rubisco carboxylation rate (t_V′cmax90_). The t_V′cmax90_ decreased significantly from ~285 s under aCO_2_ to ~147 s under eCO_2_, respectively. For mCO_2_, the values of t_A90_ and t_V′cmax90_ were 158 and 180 s, respectively, and neither parameter differed significantly from either the aCO_2_ or eCO_2_ treatments.

### 2.5. Time-Integrated Photosynthetic Limitation in Honeysuckle Leaves Under Different CO_2_ Treatments

Analysis of time-integrated limitations during photosynthetic induction revealed a highly consistent pattern across all CO_2_ treatments (Figure 5). Under the assumption that the biochemical limitation is primarily Rubisco-limited, the apparent non-stomatal component was larger than the stomatal component. Furthermore, because elevated CO_2_ altered neither S_L_V_ nor B_L_V_, the fundamental hierarchy of limitations remained unchanged. To assess the sensitivity of our limitation partitioning to the assumed biochemical mechanism, we recalculated B_L_ under an alternative scenario where the biochemical limitation was treated as RuBP regeneration limitation rather than Rubisco-limited (Supplementary Method S1). This alternative calculation yielded virtually identical results to those shown in Figure 5, with B_L_ values remaining >0.9 under all CO_2_ treatments (Supplementary Figure S1).

## 3. Discussion

Our findings indicated that eCO_2_ markedly influenced photosynthetic induction in honeysuckle leaves during the transition from low to high light. When the CO_2_ concentration increased from 420 to 820 μmol mol^−1^, *A* increased significantly, whereas g_s_ and *V*′*_cmax_* decreased. Elevated CO_2_ also reduced the time required for *A* and *V*′*_cmax_* to attain 90% of their steady-state values, indicating a faster adjustment of the photosynthetic apparatus after a sudden increase in light intensity. Limitation analysis showed that the apparent biochemical limitation (note: this is a lumped parameter because mesophyll conductance was not measured and therefore includes both carboxylation kinetics and internal CO_2_ diffusion resistance) was the dominant constraint during induction under all CO_2_ treatments, while stomatal limitation remained relatively small. These results suggest that eCO_2_ promotes photosynthetic induction in honeysuckle mainly by increasing CO_2_ availability and improving the apparent efficiency of Rubisco carboxylation, rather than by altering the dominant limitation from biochemical to stomatal processes. However, we acknowledge that our analysis did not account for mesophyll conductance, and thus the mechanistic interpretation should be treated with caution.

### 3.1. Elevated CO_2_ Enhanced Steady-State Carbon Assimilation

In this study, under high-light steady-state conditions, *A* of honeysuckle leaves increased significantly with rising external CO_2_ concentration (Figure 3). This is because eCO_2_ substantially elevated the substrate concentration for Rubisco carboxylation and effectively suppressed photorespiration. Concurrently, we observed that *V*′*_cmax_* declined progressively with increasing CO_2_ concentration during both the initial low-light phase and the high-light steady-state phase (Figure 2 and Figure 3). Recently, Tang et al. reported strikingly similar findings in *Populus tomentosa* and *Eucalyptus robusta*, where 800 µmol mol^−1^ CO_2_ treatment simultaneously enhanced steady-state *A* while significantly reducing *V_cmax_* [21]. Together with our results in honeysuckle, these findings suggest that elevated CO_2_ can enhance apparent carbon assimilation. 

### 3.2. Elevated CO_2_ Accelerated Photosynthetic Induction in Honeysuckle Leaves

In natural canopies, plants frequently experience intense light fluctuations caused by shading, wind movement, and other factors, making the rate of photosynthetic induction during low-to-high light transitions a critical determinant of transient light-use efficiency and cumulative daily carbon gain [9]. Our study revealed that eCO_2_ significantly accelerated the induction kinetics in honeysuckle (Figure 4). This acceleration effect may be partially attributed to the elevated substrate concentration under high CO_2_, which appears to alleviate the apparent biochemical limitation during the early induction phase. As suggested by previous studies [22,23,24], the time required to reach full photosynthetic capacity following a light transition is often associated with biochemical processes such as Rubisco activation. However, because we did not directly measure Rubisco activation state, RuBP regeneration, electron transport, or mesophyll conductance in the present study, we cannot attribute the observed acceleration to any specific physiological mechanism. Instead, our results indicate that, within the assumptions of the applied two-component model, the apparent non-stomatal component was alleviated more rapidly under eCO_2_. This mechanism appears consistent with the conclusions of Taylor and Long in crop species and is further corroborated by the recent data of Tang et al. in broadleaved tree species [10,21]. These collective results demonstrate that acceleration of dynamic photosynthetic responses through substrate compensation under elevated CO_2_ may not be restricted to a single taxonomic group. We therefore propose the testable hypothesis that substrate compensation represents a conserved adaptive strategy across diverse plant functional types, including crops, trees, and medicinal vines such as honeysuckle, a possibility that warrants further investigation across a broader range of species and growth conditions.

### 3.3. Photosynthetic Induction Was Mainly Constrained by Biochemical Rather than Stomatal Limitation

Within the assumptions of the applied two-component gas-exchange model, and assuming that the biochemical limitation is primarily Rubisco-limited, the time-integrated limitation analysis suggested that the apparent biochemical component consistently exceeded the stomatal component across all measured CO_2_ concentrations (Figure 5). However, it must be emphasized that our limitation analysis assumed infinite mesophyll conductance (g_m_). Because we did not measure or model g_m_, any internal CO_2_ diffusion resistance from the intercellular spaces to the chloroplast stroma would have been absorbed into the calculated biochemical component. Thus, the “biochemical limitation” we report is in fact an apparent lumped parameter that includes both true carboxylation kinetics and mesophyll diffusion limitation. Consequently, we cannot separate the contribution of mesophyll conductance from that of biochemical processes based on our current dataset. Although previous studies on crop and woody species have suggested that slow stomatal responses may cause substantial CO_2_ supply deficits during the early phase of induction, thereby making stomatal limitation a major constraint on dynamic photosynthesis [24], honeysuckle appears to adopt a distinct stomatal regulation strategy as a climbing vine. It maintained relatively consistent and effective basal stomatal aperture during the low-light pre-adaptation phase, thereby avoiding severe CO_2_ diffusional constraints at the moment of high-light appearance. This consistent limitation pattern contrasts sharply with the recent findings of Tang et al. in broadleaved tree species [21]. Elevated CO_2_ shifted the dominant limitation from stomatal to biochemical in *P. tomentosa* and *E. robusta*, whereas honeysuckle already exhibited biochemical limitation dominance under aCO_2_. Evidently, while eCO_2_ considerably accelerated the induction process in honeysuckle by enriching the substrate pool, it did not reshape the underlying limitation hierarchy. This divergence highlights strong species-specific differences in the relative contributions of physiological limitations among plant life forms under dynamic light conditions.

### 3.4. Limitations of the Photosynthetic Induction Mechanism and Implications for Future Cultivation and Quality Assessment

Although elevated CO_2_ significantly accelerated the photosynthetic induction process and enhanced instantaneous carbon gain, the persistent dominance of B_L_ indicates that eCO_2_ merely optimized substrate use efficiency within the existing physiological framework, without fundamentally removing the biochemical bottleneck of carbon assimilation. This intrinsic limitation hierarchy suggests that the photosynthetic induction accompanying *V_cmax_* downregulation may involve more complex photochemical energy partitioning as reported by Tang et al. [21]. Therefore, future studies urgently need to couple gas exchange with chlorophyll fluorescence techniques and combine quantitative analysis of photosynthetic proteins and non-structural carbohydrates to fully dissect the coordination between carbon reactions and light reactions under elevated CO_2_. From an applied production perspective, honeysuckle, as a vine with complex canopy architecture, depends critically on rapid photosynthetic response capacity to maximize cumulative daily carbon gain in heterogeneous light environments.

## 4. Materials and Methods

### 4.1. Plant Material and Culture

*Lonicera japonica* Thunb. seedlings with uniform growth were selected from a local nursery in Zhoukou. In April, all seedlings were transplanted into 8 L plastic pots containing a mixed growing substrate supplemented with slow-release NPK fertilizer to support subsequent growth. The potted plants were maintained outdoors under natural conditions at Zhoukou Normal University and irrigated once daily. During the cultivation period in April, the mean ambient temperature was approximately 16.8 °C, and the relative humidity was approximately 58%. One month later, leaf photosynthetic traits were measured under different CO_2_ concentrations as described below. Seven days before measurement, the plants were transferred to an environment-controlled greenhouse for acclimation. During acclimation, greenhouse environment conditions were maintained at a light intensity of 800 μmol m^−2^ s^−1^, relative humidity of 60%, day/night temperature of 25/20 °C, and the photoperiod was set to 14 h light/10 h dark.

### 4.2. Photosynthetic Induction Measurements Under Different CO_2_ Concentrations

To investigate the dynamics of photosynthetic induction in response to varying CO_2_ levels, gas exchange measurements were carried out with the LI-6400 Photosynthesis System (LI-COR, Inc., Lincoln, NE, USA) at three controlled chamber CO_2_ concentrations: 420, 620, and 820 μmol mol^−1^. For each treatment, the latest fully expanded leaves were selected from three or four individual *Lonicera japonica* Thunb. plants, providing independent biological replicates. Prior to high-light exposure, all sampled leaves were subjected to a 20-min induction period under low photosynthetic photon flux density (100 μmol m^−2^ s^−1^ PPFD) to ensure a stable baseline physiological condition. Following this equilibration phase, the irradiance was abruptly raised to 1200 μmol m^−2^ s^−1^ PPFD and sustained at high light for 25 min. During the entire induction process, the CO_2_ mole fraction in the reference cuvette was maintained at the designated treatment concentration, while leaf temperature was held at 25 °C and leaf-to-air vapor pressure deficit was maintained at 1–1.5 kPa. Over the full 45-min measurement period (comprising 20 min of low light and 25 min of high light), key photosynthetic parameters were logged at 15 s intervals. The initial values *A_i_* and g_si_ were derived by averaging the last three minutes of the low-light phase, whereas *A*_f_ and g_sf_ were averaged over the last 1 min of the high-light induction period.

To evaluate the biochemical limitation during photosynthetic induction, we adopted the following assumption: the biochemical limitation is primarily dominated by the slow activation of Rubisco. A previous study reported that following the transition from low light to high light, the kinetics of Rubisco carboxylation and the regeneration of its substrate (RuBP) are the main factors limiting carboxylation capacity [24]. Accordingly, we considered Rubisco activation as the primary biochemical process causing the delayed attainment of apparent maximum carboxylation rate (*V*′*_cmax_*). *V*′*_cmax_* was calculated using the following widely used equation according to previous studies [24,25,26]:

(1) $${V\prime }_{\mathrm{cmax}}\;=\;\frac{\left(A+R_{d}){(C}_{i}+K_{m}\right)}{\left(C_{i}-\Gamma^{*}\right)}$$

In this context, R_d_ denotes the light-adapted mitochondrial respiration rate, which is fixed at 1 μmol m^−2^ s^−1^ following the protocol of Luo et al. [22]. The term K_m_ represents the effective Michaelis–Menten constant for CO_2_ taken as 541.9 μmol mol^−1^ at a temperature of 25 °C, while Γ* stands for the CO_2_ compensation point when daytime respiration is excluded, with a value of 40 μmol mol^−1^. The gas-exchange-derived *V*′*_cmax_* is interpreted as the apparent maximum Rubisco carboxylation capacity, following the standard FvCB model framework and consistent with its use in recent studies [25,27,28,29,30,31]. The ‘apparent’ qualifier does not alter the biochemical identity of the parameter as the RuBP-saturated carboxylation rate.

### 4.3. Photosynthetic Limitations Analysis During Photosynthetic Induction Process

The total deviation of *A* from its steady-state value (d*A*) is decomposed into a stomatal component (d*A*_s_) and a biochemical component (d*A*_b_). However, this framework assumes infinite mesophyll conductance (g_m_). Since we did not measure or model g_m_, the calculated biochemical component likely includes internal CO_2_ diffusion resistance from the intercellular spaces to the chloroplast stroma, in addition to true carboxylation kinetics (Rubisco activation and RuBP regeneration). Therefore, the biochemical component in our analysis should be interpreted as an apparent parameter, rather than a purely biochemical term. The decomposition is expressed following [25] as below:

(2) $$dA={dA}_{s}+{dA}_{b}=\frac{\partial A}{{\partial g}_{\mathrm{sc}}}dg_{\mathrm{sc}}+\frac{\partial A}{{\partial V\prime }_{\mathrm{cmax}}}{dV\prime }_{\mathrm{cmax}}$$

In this context, g_sc_ is the stomatal conductance to CO_2_, obtained by dividing g_s_ by 1.6, and *V′_cmax_* is the apparent maximum carboxylation rate, which is influenced by both Rubisco kinetics and mesophyll conductance. The variations dg_sc_ and d*V*′*_cmax_* are computed as the deviations between the measured values and the steady-state values reached at the end of the induction period. The partial derivatives of the assimilation rate *A* with respect to g_sc_ and *V*′*_cmax_* are evaluated as shown below:

(3) $$\frac{\partial A}{{\partial g}_{\mathrm{sc}}}=\frac{\frac{A}{g_{\mathrm{sc}}^{2}}\left(({V\prime }_{\mathrm{cmax}}-R_{d}\right)-A)}{({V\prime }_{\mathrm{cmax}}-R_{d})(\frac{1}{g_{\mathrm{sc}}})+(C_{a}+K_{m})-2(\frac{1}{g_{\mathrm{sc}}})A}$$

(4) $$\frac{\partial A}{{\partial V\prime }_{\mathrm{cmax}}}=\frac{\left(C_{a}-\Gamma^{*}\right)-A(\frac{1}{g_{\mathrm{sc}}})}{({V\prime }_{\mathrm{cmax}}-R_{d})(\frac{1}{g_{\mathrm{sc}}})+(C_{a}+K_{m})-2(\frac{1}{g_{\mathrm{sc}}})A}$$

Based on the common assumption in this framework that the biochemical limitation primarily reflects Rubisco activation (i.e., changes in *V′_cmax_*), time-integrated photosynthetic limitations imposed by stomatal (S_L_V_) and biochemical components (B_L_V_) were calculated as follows [25]:

(5) $$S_{L\_V}=\frac{\int dA_{s}dt}{\int dA_{s}dt+\int dA_{b}dt}$$

(6) $$B_{L\_V}=1-S_{L\_V}$$

To test the sensitivity of our limitation analysis to the interpretation of the biochemical component, we additionally calculated the integrated limitations under the alternative assumption that the biochemical limitation was RuBP regeneration limitation (rather than Rubisco activation; see Supplementary Materials for details). The results under this alternative scenario were nearly identical to those based on the Rubisco-activation assumption, with apparent B_L_ values consistently exceeding 0.9 across all CO_2_ treatments (Supplementary Figure S1). This consistency confirms that our qualitative conclusions are robust to the specific biochemical assumption, though it does not resolve the mechanistic ambiguity regarding the underlying biochemical steps.

### 4.4. Dynamic Kinetics of Photosynthetic Parameters

As proposed by Mott and Woodrow [22], the dynamic change in photosynthetic parameters was described by the following equation:

(7) $$X\left(t\right)=X_{f}+(X_{i}-X_{f})e^{\frac{-t}{\tau }}$$

Here, X_i_ and X_f_ represent the initial and final values of photosynthetic parameters during photosynthetic induction process. τ is the fitted time constant. The goodness of fit was evaluated using the coefficient of determination (R^2^) for each fitted curve (mean R^2^ values are reported in Figure S2). The time to reach 90% induction state was determined using τ as follows:

(8) $$t_{90}=-\tau *\ln\left(0.1\right)$$

### 4.5. Statistical Analysis

All statistical analyses were performed using R software (version 4.2.1). Given the small sample size (*n* = 3 or 4 per treatment), which limits the diagnostic power of normality (Shapiro–Wilk) and homogeneity of variance (Levene’s) tests, we adopted permutation-based tests for all comparisons to avoid distributional assumptions (lmPerm and coin packages, with 10,000 random permutations). For each variable, an overall permutation F-test (aovp function) was first applied to evaluate the effect of treatment. To address the issue of multiple testing across variables, the *p*-values were adjusted using the Benjamini–Hochberg false discovery rate (FDR) method. This step served to identify which of the parameters were genuinely responsive to treatment at the overall level. For those variables that showed a significant global treatment effect, pairwise comparisons between the three treatment groups were conducted using permutation *t*-tests. To control the family-wise error rate within each variable, the resulting *p*-values were adjusted using the Bonferroni–Holm sequential correction method. This two-tiered strategy ensured that FDR control was applied to the screening of responsive variables, while FWER control was applied to the precise localization of between-group differences within each significant variable.

## 5. Conclusions

This study demonstrates that, although eCO_2_ enhanced the net photosynthetic rate of honeysuckle under high light, elevated CO_2_ also markedly shortened the photosynthetic induction time during the transition from low to high light, accelerated the apparent activation of Rubisco, and improved transient carbon acquisition under dynamic light conditions. However, photosynthetic induction was consistently dominated by biochemical limitation, whereas stomatal limitation remained negligible. It is important to emphasize that our limitation analysis did not account for g_m_; therefore, the so-called “biochemical limitation” is in fact a lumped apparent parameter that includes internal CO_2_ diffusion resistance from the intercellular spaces to the chloroplast stroma, rather than a purely biochemical term. Overall, eCO_2_ enhanced honeysuckle’s capacity to utilize light during induction but did not alter the limitation hierarchy centered on biochemical lags.

## Figures and Tables

**Figure 1:**
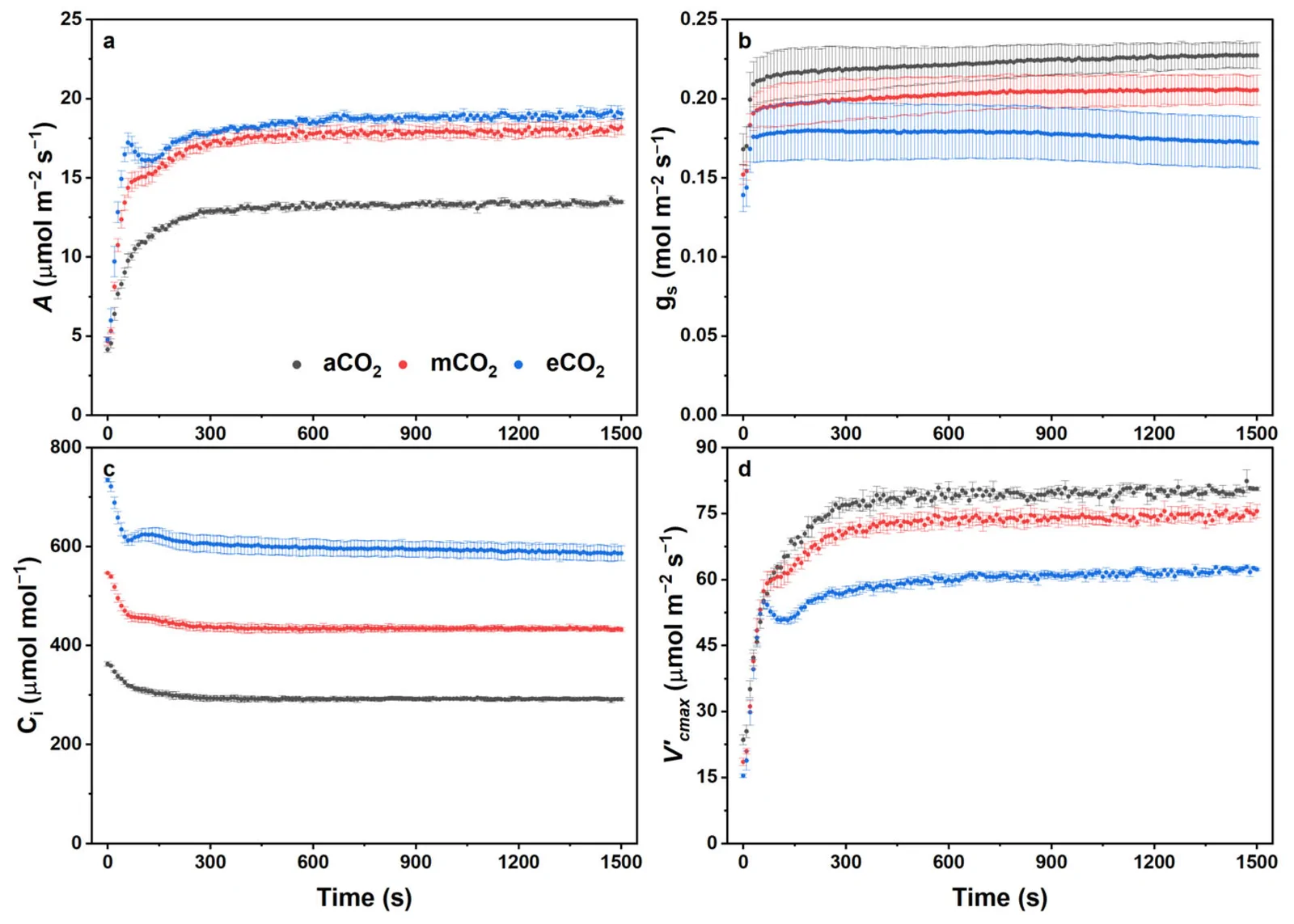
Dynamic responses of photosynthetic parameters in honeysuckle (*Lonicera japonica*) leaves to an abrupt light increase from 100 to 1200 µmol m^−2^ s^−1^ during photosynthetic induction under three CO_2_ concentrations: 420 μmol mol^−1^ (ambient, aCO_2_), 620 μmol mol^−1^ (medium, mCO_2_), and 820 μmol mol^−1^ (elevated, eCO_2_). Plots show: (**a**) *A* (net CO_2_ assimilation); (**b**) g_s_ (stomatal conductance to water vapor); (**c**) C_i_ (intercellular CO_2_ concentration); (**d**) *V*′*_cmax_* (apparent maximum Rubisco carboxylation rate). Data are means ± SE (*n* = 3 for aCO_2_ and mCO_2_, *n* = 4 for eCO_2_).

**Figure 2:**
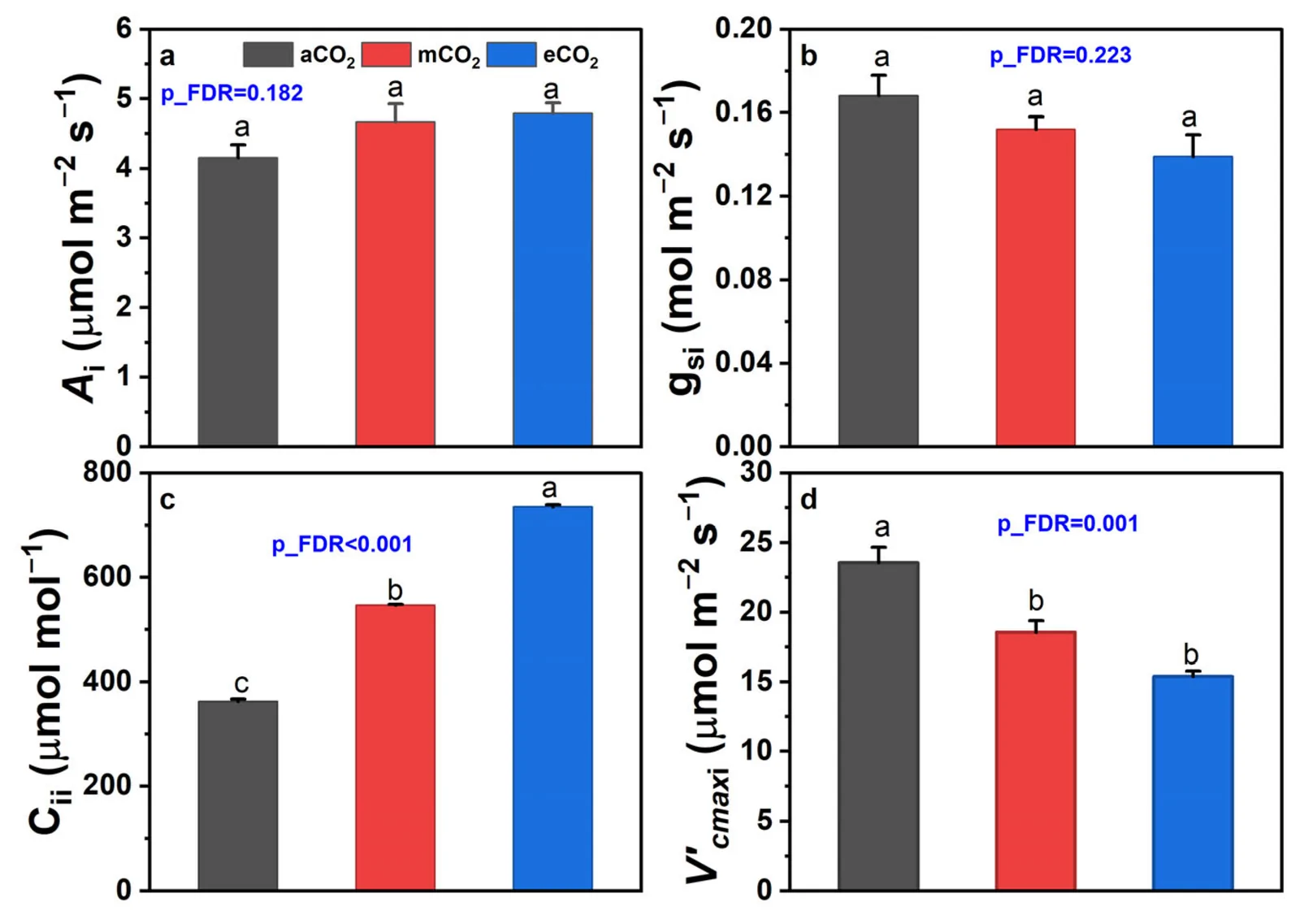
Photosynthetic parameters of honeysuckle (*Lonicera japonica*) leaves measured at the end of the low-light phase during photosynthetic induction under three CO_2_ concentrations: 420 μmol mol^−1^ (ambient, aCO_2_), 620 μmol mol^−1^ (medium, mCO_2_), and 820 μmol mol^−1^ (elevated, eCO_2_). (**a**) *A*_i_, (**b**) g_si_, (**c**) C_ii_, and (**d**) *V*′*_cmax_*_i_ represent the steady-state net photosynthetic rate, stomatal conductance to H_2_O, intercellular CO_2_ concentration, and apparent maximum carboxylation rate, respectively, averaged over the last 3 min of the low-light condition (100 µmol m^−2^ s^−1^). Different letters indicate significant differences between treatments. Overall *p*-values for each parameter were corrected using the Benjamini–Hochberg FDR method (p_FDR < 0.05). Data are means ± SE (*n* = 3 for aCO_2_ and mCO_2_, *n* = 4 for eCO_2_).

**Figure 3:**
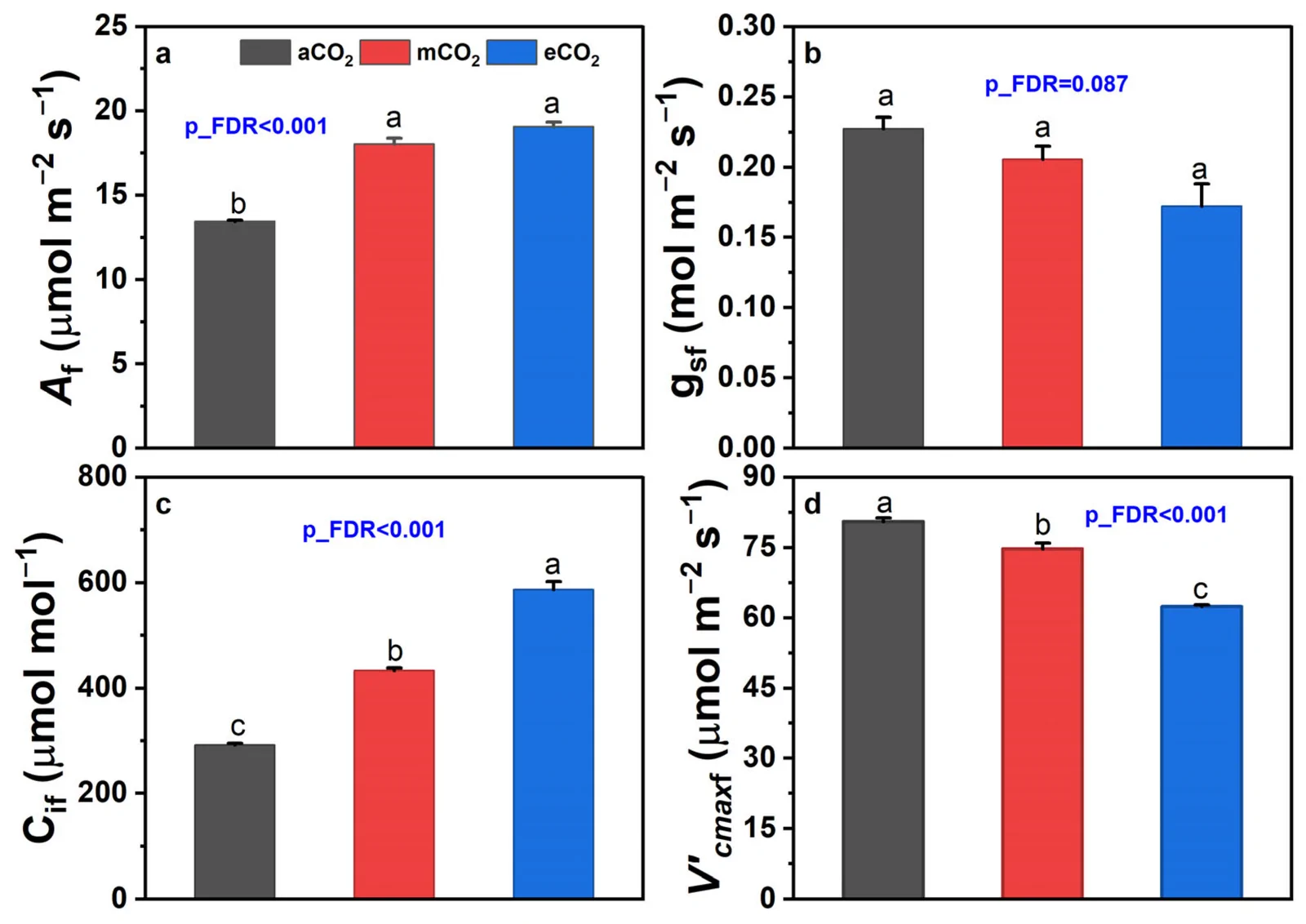
Photosynthetic parameters of honeysuckle (*Lonicera japonica*) leaves measured at the end of the high-light phase during photosynthetic induction under three CO_2_ concentrations: 420 μmol mol^−1^ (ambient, aCO_2_), 620 μmol mol^−1^ (medium, mCO_2_), and 820 μmol mol^−1^ (elevated, eCO_2_). (**a**) *A*_f_, (**b**) g_sf_, (**c**) C_if_, and (**d**) *V*′*_cmax_*_f_ represent the steady-state net photosynthetic rate, stomatal conductance to H_2_O, intercellular CO_2_ concentration, and apparent maximum carboxylation rate, respectively, averaged over the last 1 min of the high-light condition (1200 µmol m^−2^ s^−1^). Different letters indicate significant differences between treatments. Overall *p*-values for each parameter were corrected using the Benjamini–Hochberg FDR method (p_FDR < 0.05). Data are means ± SE (*n* = 3 for aCO_2_ and mCO_2_, *n* = 4 for eCO_2_).

**Figure 4:**
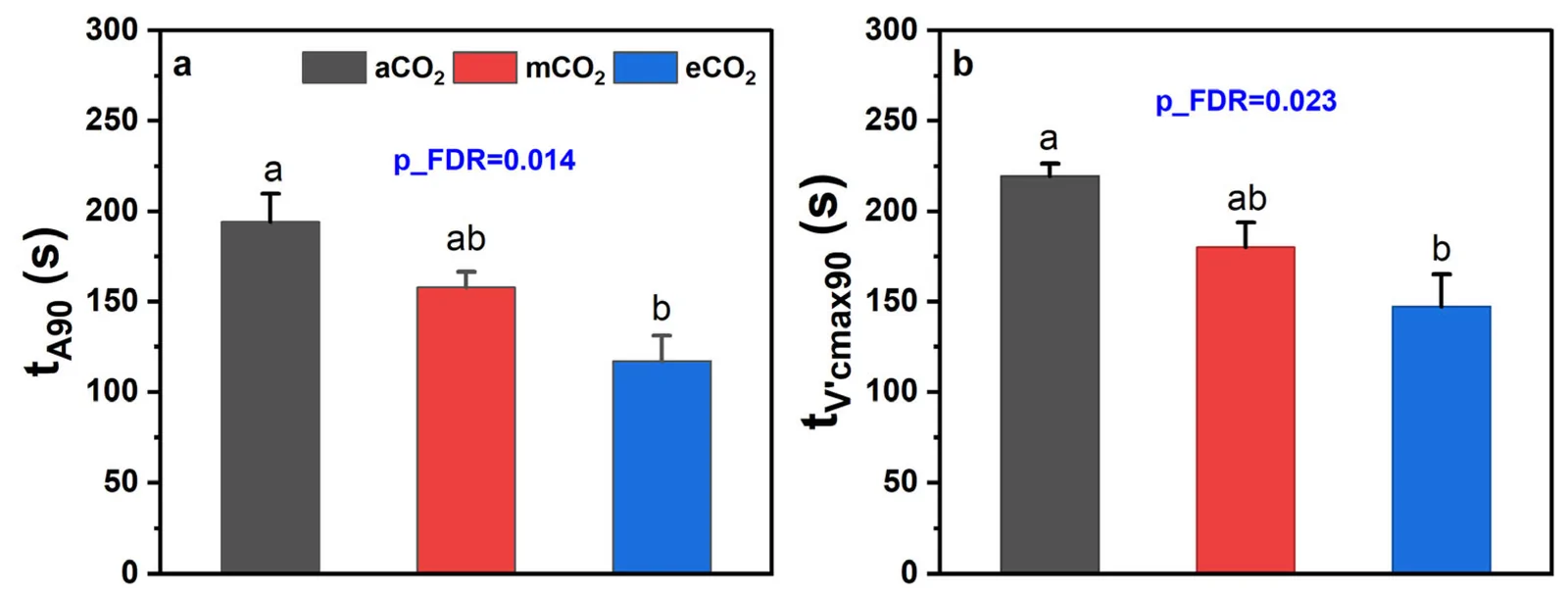
Induction dynamics of photosynthetic parameters in honeysuckle (*Lonicera japonica*) leaves under three CO_2_ concentrations: 420 μmol mol^−1^ (ambient, aCO_2_), 620 μmol mol^−1^ (medium, mCO_2_), and 820 μmol mol^−1^ (elevated, eCO_2_). (**a**) t_A90_: time to achieve 90% of the steady-state net photosynthetic rate (*A*); (**b**) t_V′cmax90_: time to achieve 90% of the apparent maximum carboxylation rate (*V*′*_cmax_*). Different letters indicate statistically significant differences among treatments. Overall *p*-values for each parameter were corrected using the Benjamini–Hochberg FDR method (p_FDR < 0.05). Data are presented as means ± SE (*n* = 3 for aCO_2_ and mCO_2_, *n* = 4 for eCO_2_).

**Figure 5:**
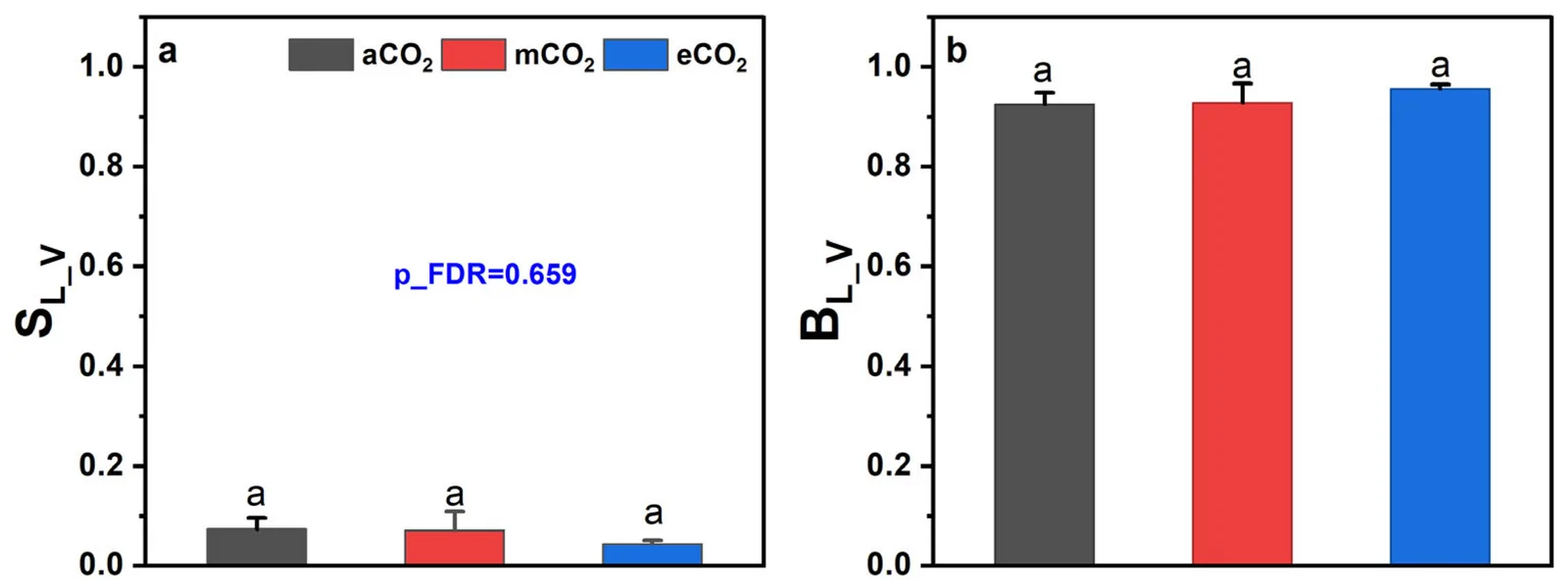
The time-integrated limitations in honeysuckle (*Lonicera japonica*) leaves during photosynthetic induction under three CO_2_ concentrations: 420 μmol mol^−1^ (ambient, aCO_2_), 620 μmol mol^−1^ (medium, mCO_2_), and 820 μmol mol^−1^ (elevated, eCO_2_). (**a**) Time-integrated stomatal limitation based on Rubisco activation limitation (S_L_V_); (**b**) time-integrated biochemical limitation based on Rubisco activation limitation (B_L_V_) in response to photosynthetic induction. The same lowercase letters indicate no significant differences among the three CO_2_ treatments for each limitation type. Overall *p*-values for each limitation type were corrected using the Benjamini–Hochberg FDR method (p_FDR < 0.05). Since SL = 1 − BL, both limitation types share identical *p*-values and significance patterns. Within each panel, the same lowercase letters indicate no significant differences among the three CO_2_ treatments. Data are presented as means ± SE (*n* = 3 for aCO_2_ and mCO_2_, *n* = 4 for eCO_2_).

## Data Availability

The data are contained within the article.

## Supplementary Materials

The following supporting information can be downloaded at: https://www.mdpi.com/article/10.3390/plants15192929/s1, Figure S1: The time-integrated limitations in honeysuckle (*Lonicera japonica*) leaves during photosynthetic induction under three CO_2_ concentrations: 420 μmol mol^−1^ (ambient, aCO_2_), 620 μmol mol^−1^ (medium, mCO_2_), and 820 μmol mol^−1^ (elevated, eCO_2_); Figure S2: Goodness-of-fit statistics for Equation 7 fitted to the measured induction curves of net photosynthetic rate (*A*) and apparent maximum carboxylation rate (V′_cmax_) under three CO_2_ treatments: 420 μmol mol^−1^ (ambient, aCO_2_), 620 μmol mol^−1^ (medium, mCO_2_), and 820 μmol mol^−1^ (elevated, eCO_2_); Method S1: RuBP regeneration-based limitation analysis during photosynthetic induction [32].
