# Supplementary material for: Dynamic Responses of Photosynthetic Parameters in Lonicera japonica Leaves During Photosynthetic Induction Under Elevated CO2 Concentrations
Source: Plants (Basel). 2026 Sep 24;15(19):2929. doi: 10.3390/plants15192929 (PMC13667207; doi:10.3390/plants15192929)
Supplement: Supplementary file 1 [file plants-15-02929-s001.zip › plants-4542967-supplementary.pdf]

Supplementary Materials

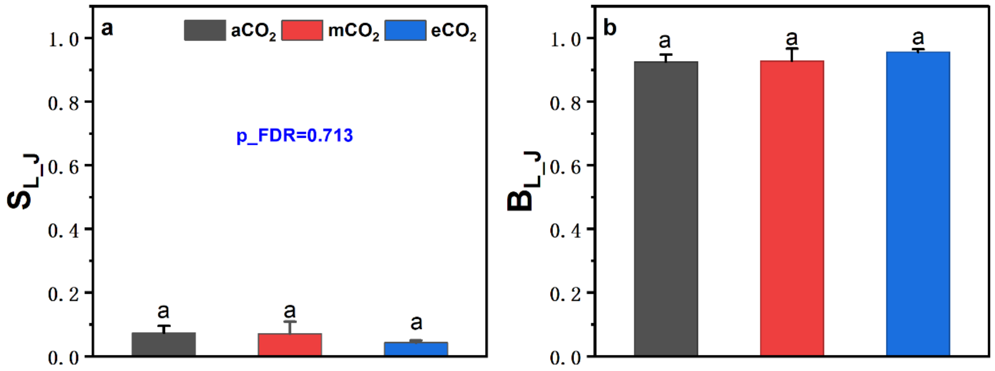

Figure S1. The time-integrated limitations in honeysuckle (*Lonicera japonica*) leaves during photosynthetic induction under three CO_2 concentrations: 420 μmol mol^{-1} (ambient, aCO_2), 620 μmol mol^{-1} (medium, mCO_2), and 820 μmol mol^{-1} (elevated, eCO_2). (a) time-integrated stomatal limitation based on RuBP regeneration limitation (S_{L_J}); (b) time-integrated biochemical limitation based on RuBP regeneration limitation (B_{L_J}) in response to photosynthetic induction. The same lowercase letters indicate no significant differences among the three CO_2 treatments for each limitation type. Overall p-values for each limitation type were corrected using the Benjamini–Hochberg FDR method ($p\_FDR < 0.05$). Since SL = 1 − BL, both limitation types share identical P-values and significance patterns. Within each panel, the same lowercase letters indicate no significant differences among the three CO_2 treatments. Data are presented as means ± SE (n = 3 for aCO_2 and mCO_2, n = 4 for eCO_2).

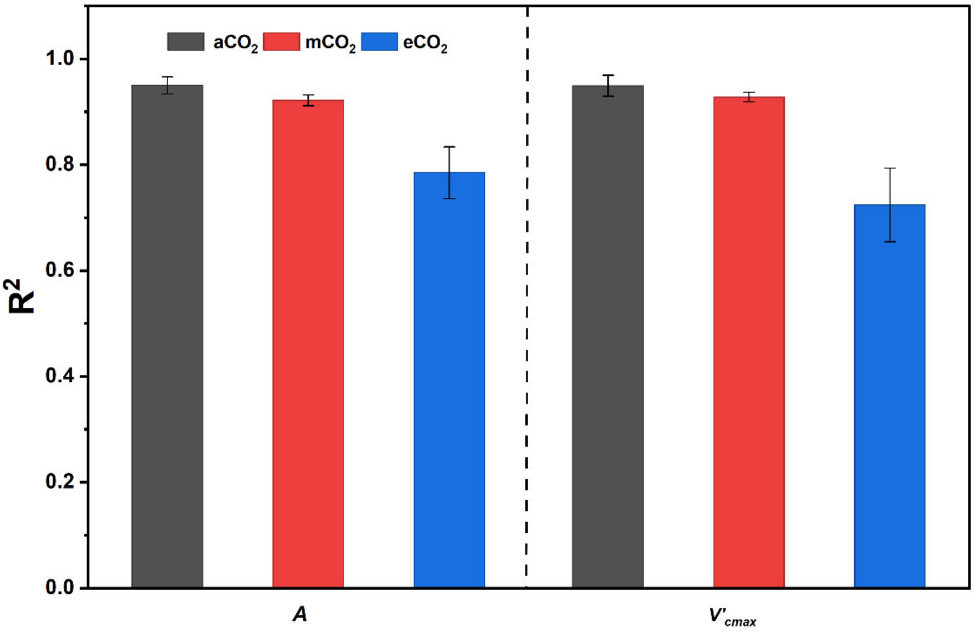

Figure S2. Goodness-of-fit statistics for Equation 7 fitted to the measured induction curves of net photosynthetic rate (*A*) and apparent maximum carboxylation rate (V'_{cmax}) under three CO_2 treatments: 420 μmol mol^{-1} (ambient, aCO_2), 620 μmol mol^{-1} (medium, mCO_2), and 820 μmol mol^{-1} (elevated, eCO_2). Values represent the coefficient of determination (R^2) obtained from the curve-fitting procedure. Data are presented as means ± SE (n = 3 for aCO_2 and mCO_2, n = 4 for eCO_2).

Method S1 RuBP regeneration-based limitation analysis during photosynthetic induction

To evaluate the biochemical limitation during photosynthetic induction, we focused on the regeneration of ribulose-1,5-bisphosphate (RuBP) as the primary biochemical process constraining the attainment of maximum carboxylation capacity under fluctuating light. Following the transition from low light to high light, the kinetics of RuBP regeneration are driven by electron transport rate (*J*), which determines the rate at which the Calvin–Benson cycle can resupply RuBP to Rubisco carboxylation sites [32]. Accordingly, we quantified the biochemical limitation associated with RuBP regeneration directly using the electron transport rate (*J*). The apparent electron transport rate (*J′*) was calculated from the measured gas-exchange parameters as follows:

$$J' = \frac{(A + R_d)(4C_i + 8\Gamma^*)}{C_i - \Gamma^*} \quad \text{(S1)}$$

In this context, R_d denotes the light-adapted mitochondrial respiration rate, which is fixed at 1 μmol m^{-2} s^{-1}. Γ^* stands for the CO_2 compensation point when daytime respiration is excluded, with a value of 40 μmol mol^{-1}. C_i represents intercellular CO_2 concentration.

The biochemical component in our analysis is represented by the apparent electron transport rate, which serves as a proxy for RuBP regeneration capacity. The decomposition is expressed as below:

$$dA = dA_s + dA_b = \frac{\partial A}{\partial g_{sc}} dg_{sc} + \frac{\partial A}{\partial J'} dJ' \quad \text{(S2)}$$

In this context, g_{sc} is the stomatal conductance to CO_2, obtained by dividing gs by 1.6, and J' is the apparent electron transport rate. The variations dg_{sc} and dJ' are computed as the deviations between the measured values and the steady-state values reached at the end of the induction period. The partial derivatives of the assimilation rate A with respect to g_{sc} and J' are evaluated as shown below:

$$\frac{\partial A}{\partial g_{sc}} = \frac{\frac{A}{g_{sc}^2}((J' - R_d) - A)}{(J' - 4R_d)(\frac{1}{g_{sc}}) + (4C_a + 8\Gamma^*) - 8(\frac{1}{g_{sc}})A} \quad \text{(S3)}$$

$$\frac{\partial A}{\partial J'} = \frac{(C_a - \Gamma^*) - A(\frac{1}{g_{sc}})}{(J' - 4R_d)(\frac{1}{g_{sc}}) + (4C_a + 8\Gamma^*) - 8(\frac{1}{g_{sc}})A} \quad \text{(S4)}$$

Based on the assumption that the biochemical limitation primarily reflects RuBP regeneration capacity (i.e., changes in electron transport rate), time-integrated photosynthetic limitations imposed by stomatal (S_{L_J}) and biochemical components (B_{L_J}) were calculated as follows:

$$S_{L\_J} = \frac{\int dA_s\, dt}{\int dA_s\, dt + \int dA_b\, dt} \quad \text{(S5)}$$

$$B_{L\_J} = 1 - S_{L\_J} \quad \text{(S6)}$$
